# Passive lower leg stiffness changes during a 10 k run

**DOI:** 10.3389/fspor.2025.1662460

**Published:** 2025-10-21

**Authors:** Anh Phong Nguyen, Laurent Bosquet, Philippe Mahaudens, Christine Detrembleur

**Affiliations:** ^1^ Université Catholique de Louvain, Institut de Recherche Expérimentale et Clinique, Neuromusculoskeletal Lab, Brussels, Belgium; ^2^ Université de Poitiers, UFR-STAPS, Move Lab, Poitiers, France

**Keywords:** myotonometry, running, tendon, myofascial tissue, biomechanics

## Abstract

The primary aim of this study was to assess lower limb stiffness in healthy runners over the course of a 10 km race at a competitive pace, with a secondary focus on investigating the tissue-specific changes in stiffness of lower limb structures during the running event. Twenty participants (3 women and 17 men) were recruited for this study. Passive stiffness measurements were assessed on the Achilles tendon, medial gastrocnemius, patellar tendon, and plantar fascia using a myotonometry device. Measurements were recorded before, during, and after the 10 k run. Our findings reveal a moderate decrease of 7% (*p* = 0.044, hedge's *g* = 0.51) of the plantar fascia's passive stiffness at the end of the trial. Contrary to the initial hypothesis, which predicted a decrease in Achilles tendon stiffness during the race, our study findings indicate that the stiffness of the Achilles tendon, patellar tendon, and medial gastrocnemius remained constant. The plantar fascia finding is novel as few studies explore foot myofascial tissue. Further investigation is warranted to elucidate the mechanisms behind the differential response of the plantar fascia compared to other tissues during prolonged running activity.

## Introduction

Running is widely recognized as one of the most accessible and beneficial forms of physical activity (1). Biomechanical factors have been extensively studied with the aim of improving running techniques for injury or performance (2). Concerning running performance, biomechanical metrics, such as passive stiffness of the lower limb tissue, are increasingly recognized as valuable for enhancing performance, particularly through improvements in running economy and efficiency (3, 4).

Passive stiffness refers to the inherent resistance of a tissue to deformation while at rest (5, 6). In the context of sport, high passive stiffness of the lower limb has been consistently associated to better running performance (7–9). Bohm et al. (10), for instance, reported that tendons serve not only as energy reservoirs, but also facilitate optimal shortening of muscle contractile elements during tendon elongation (10). Lower limb passive stiffness plays a pivotal role in two fundamental phenomena: power amplification and power attenuation (11). The former denotes the limb's capacity to generate force surpassing the one only produced by the contractile part, owing to elastic energy stored not only in tendons but also in other passive structures such as muscle fascicles and aponeuroses. Conversely, power attenuation enables the tendon to elongate and temporarily store elastic energy during rapid movements, thereby preventing excessive muscle lengthening while still generating an appropriate force output (12).

To assess the passive stiffness of musculoskeletal structures, a variety of methodologies have been explored. Laboratory-based approaches such as the sinusoidal oscillatory method and rheometry provide precise quantification but remain costly and impractical for field use (13). Imaging-based approaches, particularly ultrasound elastography, have gained traction. Shear wave elastography applies Young's modulus principles to evaluate tissue stiffness by measuring wave propagation speed and has demonstrated good reliability for tendon assessment (14, 15). B-mode ultrasound combined with dynamometry has also been used to estimate tendon stiffness and strain during controlled loading, although this method is technically demanding (16, 17). More recently, myotonometry has emerged as a convenient, portable, and field-applicable tool for assessing superficial muscles and tendons. This technique, which measures tissue oscillation following a brief mechanical impulse, has been shown to correlate well with elastography and rheometry (5). With its accessibility and ease of use, myotonometry represents a valuable addition for researchers and clinicians when evaluating myofascial stiffness in both laboratory and ecological settings (18).

With the introduction of these more accessible technologies in the field, researchers can now observe the rheological behaviors of tissues *in vivo* and during sporting events. This capability presents an opportunity to gain insights into the biomechanical characteristics of runners' tissues, not only for enhancing performance but also for injury prevention. For example, Nguyen et al. (19) observed a potential decrease in the Achilles tendon and gastrocnemius muscle stiffness during an incremental running trial. The authors stated that the explanation could be multiple and included fatigue, incremental speed or prolonged run (19). Previous investigations into stiffness changes during prolonged running have reported mixed results. Some studies have reported no change in Achilles tendon stiffness after steady-state or marathon protocols (20, 21), while others observed reductions following more intense or longer-duration efforts (22). However, to our knowledge, no study has systematically examined the variation in tendon stiffness before, during, and after a continuous running protocol at a competitive pace.

Therefore, the aims of this study were to investigate the change in (a) the Achilles tendon (AT) stiffness, as well as (b) the patellar tendon (PT), the medialis gastrocnemius (MG), and the plantar fascia (PF), in healthy runners during a 10 km race at a competitive pace, representing the prolonged run without the influence of high speed. We believe that a 10 k protocol represented better the ecological situation of runners. We hypothesized that lower limb stiffness will decrease during a prolonged run (3).

## Methods

The study was conducted at the Catholic University of Louvain (UCLouvain) in Belgium and received the approval of the local Ethics Committee (CEHF-No: B403201523492).

### Population

Twenty participants were enrolled in the study. The inclusion criteria were (1) being 18 years old or above and (2) have a minimum of one year regular running experience. They were also required to demonstrate the ability to run a distance of 15 kilometers per week and be familiar with their maximum aerobic speed (MAS). Exclusion criteria included any history of lower limb musculoskeletal pathologies within the past 12 months or neurological pathologies.

### Procedure

Participants received a complete information about the objectives of the study, the protocol, and the protection of their personal data before providing their written consent. Demographic information including age, sex, body weight, body height, medical history, frequency and volume of weekly training, years of running experience, and MAS were collected before the experimental session. For participants who were unaware of their MAS, a continuous multistage exercise test, inspired by the University of Montreal Track Test (Vameval test), was conducted at least one week prior to the experimental session. The test involved running on a marked track with cones placed every 20 meters. Participants began at a speed of 8 km·h^−1^, with the speed increasing by 0.5 km·h^−1^ every minute until exhaustion.

During this session, participants completed a 10 km run on a 200 m indoor track, structured as ten consecutive 1 km bouts. At the end of each kilometer, passive stiffness of AT, MG and PT was assessed while participants lay prone, with their running shoes on. Each measurement lasted approximately 30 s. At the 5 km mark, shoes were removed to allow access to the PF, extending the procedure to about 90 s before resuming the run. PF measurements were therefore performed at baseline, 5 km, and 10 km with participants barefoot. This protocol was designed to balance the need for repeated assessments with the feasibility of completing a continuous 10 km run. Participants initiated the run at 75%–85% of their MAS and were instructed to maintain a constant pace. Lap times were provided every 500 m, with corrective feedback, e.g., “run slower” or “increase pace”, to ensure stable running speed across the trial.

### Material

Stiffness was measured with the MyotonPro device (Myoton AS, Tallinn, Estonia), a portable and non-invasive tool designed to assess the biomechanical properties of soft tissues. This device utilizes mechanical deformation to evaluate the stiffness (in N.m^−1^) of lower limb muscles and tendons. The MyotonPro probe was applied perpendicularly to the skin surface at the predefined anatomical landmarks, with light pre-compression, following standardized recommendations (5). Subsequently, the acceleration signal was processed to generate an oscillation curve, from which the stiffness was calculated (6). A previous study has reported moderate-to-excellent intra- and inter-rater reliability for plantar flexor muscle tissue, including the achilles tendon, with intraclass correlation coefficients ranging from 0.73 to 0.96, and minimal detectable changes between ∼10% and 25% depending on the site and condition (19). Measurements using the MyotonPro device were conducted both before and during the test to assess changes in tissue stiffness by an experienced physiotherapist, i.e., 10 years of experience.

### Measurement

**Figure 1:**
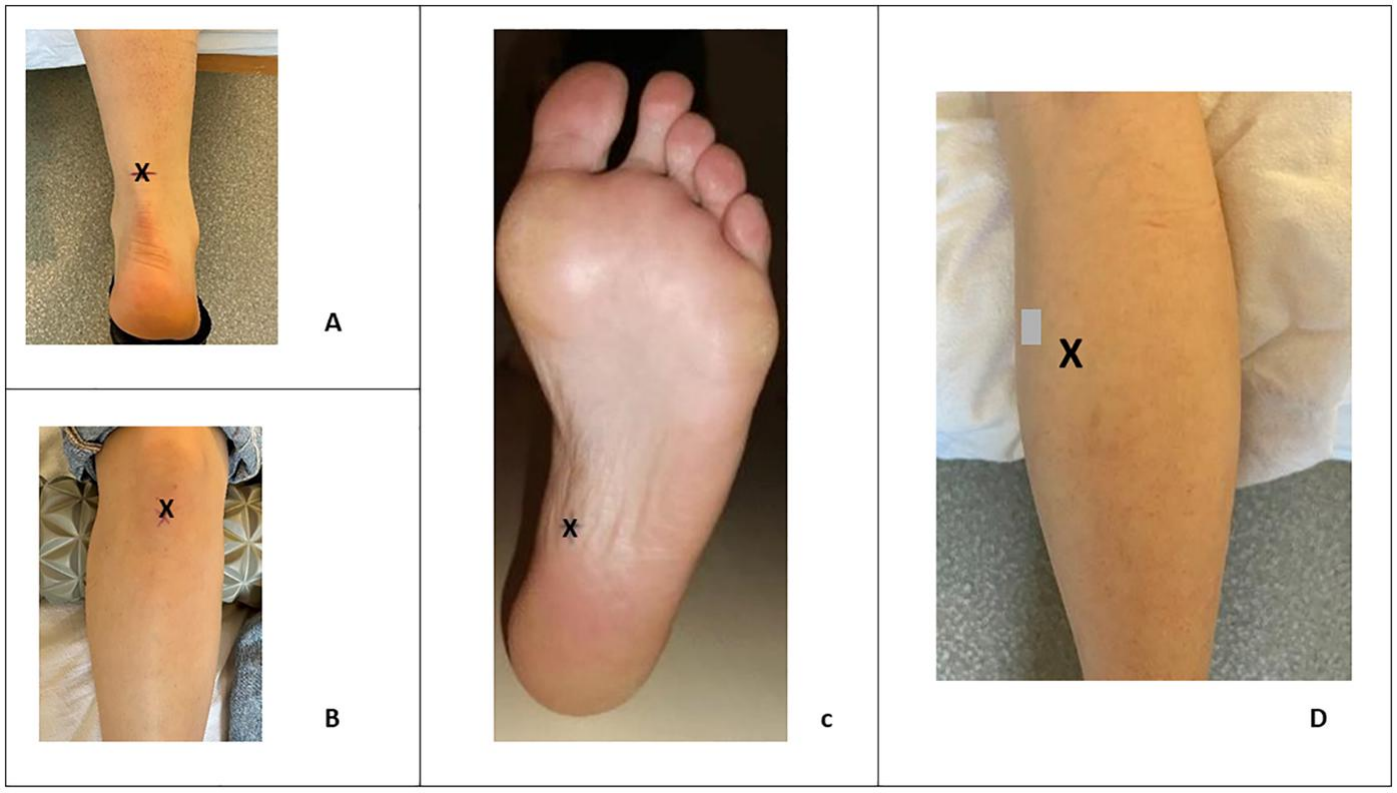
Location (X) of stiffness measurement in the Achilles tendon **(A)**, the patellar tendon **(B)**, the plantar fascia **(C)** and on the gastrocnemius medialis **(D)**.

All measurement points were anatomically identified. The Achilles tendon stiffness was recorded 8 cm above its insertion at the calcaneus. The stiffness of the MG was measured at the belly of the muscle. For the PT, stiffness was assessed at the midpoint between the patella and the anterior tibial tuberosity. As for the PF, stiffness measurement was performed at its proximal insertion on the calcaneus. During measurements of the AT, MG and PF, the participants were positioned in a prone position on an examination table, with their ankle positionned at the edge of the table and their feet hanging freely in the air. Conversely, for measurements of the PT, the participants were placed in a supine position on the examination table, with a cushion positioned under the popliteal fossa (Figure 1).

### Statistical analysis

Statistical analyses were conducted using MedCalc® Statistical Software version 23.0.8 (MedCalc Software Ltd, Ostend, Belgium; 2024). Normality of the data was assessed using the Shapiro–Wilk test. A one-way repeated measures ANOVA was carried out to test the null hypothesis of an absence of effect of time on passive stiffness. Where necessary, a Tukey *post-hoc* analysis was applied. Additionally, Hedge's g effect sizes were calculated to quantify the magnitude of differences observed, with values interpreted as small (0.2), medium (0.5), and large (0.8) effects. The significance level was set at alpha = 0.05.

## Results

Twenty-two healthy volunteers were included in this study. Their characteristics are presented in Table 1. No runner had to stop or significantly lower his/her pacing during the trial. The measurement at each kilometer lasted leds than a minute. The complete measurement value can be found in Table 2.

**Table 1:** Descriptive data of participants.

	mean (standard deviation)
Men/women (*n*)	18/4
Age (years)	26.5 (8.6)
Height (cm)	178 (8.6)
Weight (kg)	69.9 (12.5)
Km per week	25.4 (8.1)
Session per week	3.0 (1.0)
Running experience (years)	7.8 (9.4)
Maximal aerobic speed (km h^−1^)	16.6 (1.6)

Data are expressed in mean (standard deviation).

**Table 2:** Mean and standard deviation of passive stiffness measurement during the 10 k run.

Distance	Achilles tendon		Patellar tendon		Gastrocnemius medialis		Plantar fascia	
	Mean	sd	Mean	sd	Mean	sd	Mean	sd
0 (Start)	857.6	120.7	591.2	99.1	339.5	66.2	431.3	60.8
1	824.2	136.2	590.6	79.8	370.4	80.7		
2	824.8	150.8	583.3	88.3	360.7	60.0		
3	828.5	151.2	575.4	96.8	364.0	78.5		
4	817.1	146.2	580.4	86.4	377.8	95.1		
5	806.5	139.1	578.1	84.3	359.6	82.1	401.5	48.5
6	821.6	159.3	551.3	100.2	352.8	72.5		
7	816.3	146.2	560.4	104.8	346.6	76.0		
8	826.8	133.2	544.5	127.4	342.0	61.5		
9	811.1	133.0	581.4	100.0	339.0	63.8		
10	808.9	140.2	553.1	119.7	331.9	60.0	397.6	42.0

sd, standard deviation.

**Figure 2:**
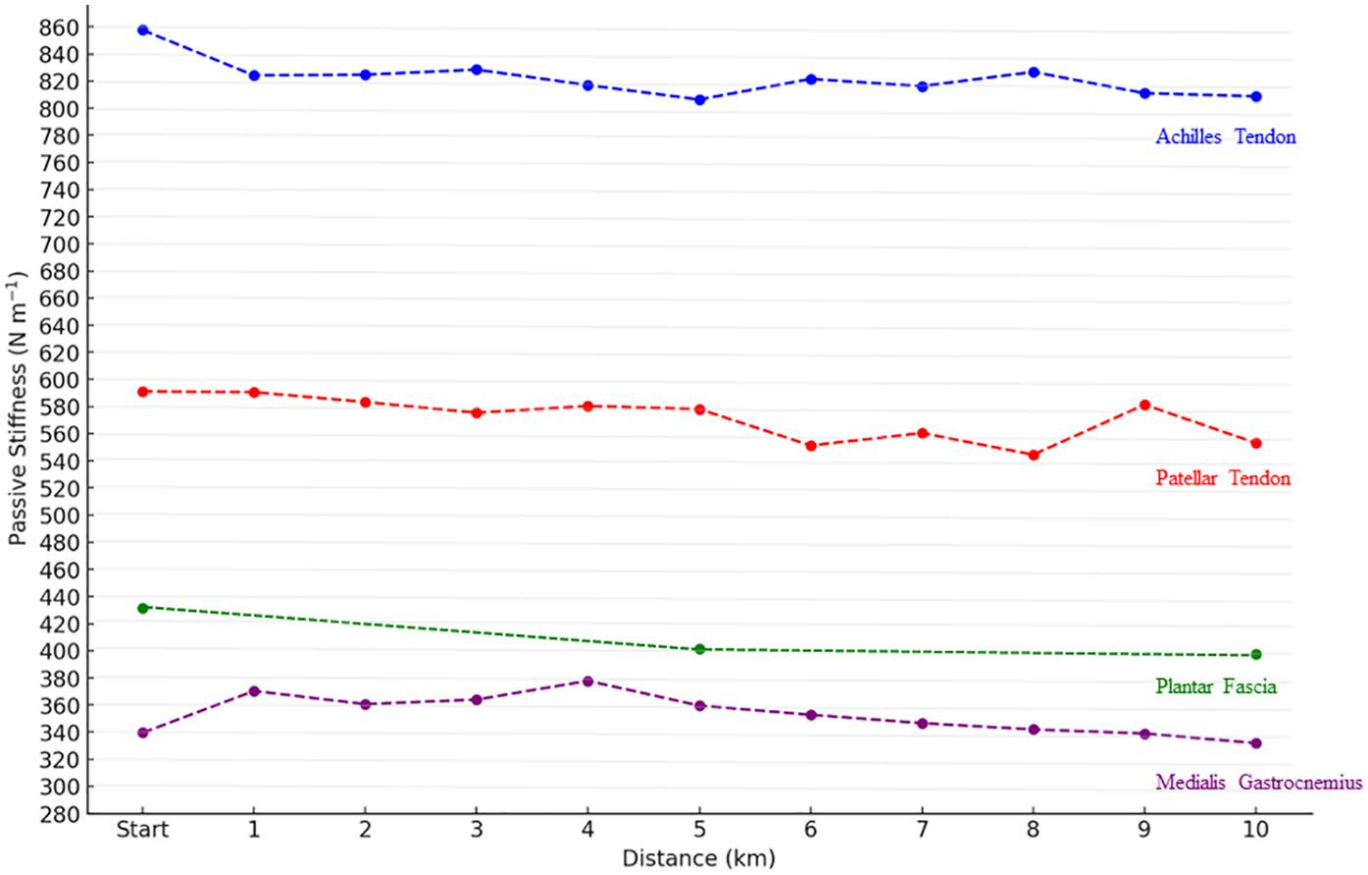
Evolution of lower limb stiffness (N·m^−1^) throughout the 10 km run. Start mean kilometer 0. Data point represent mean values. Significant differences are indicated by * in comparison with Baseline (0 km), specifically for plantar fascia stiffness at the 5th and 10th kilometers.

The analysis of AT stiffness using repeated measures ANOVA revealed no significant effect of distance [*F*(10, 427) = 0.37, *p* = .959, *η*^2^ = .009] with values remaining stable throughout the 10 km run. Similarly, MG stiffness did not change significantly across distance [*F*(10, 407) = 1.51, *p* = .134, *η*^2^ = .036] and PT stiffness also showed no effect of distance [*F*(10, 400) = 1.03, *p* = .418, *η*^2^ = .025]. In contrast, a significant effect of distance was found for PF stiffness [*F*(2, 112) = 5.06, *p* = .008, *η*^2^ = .083]. *post hoc* Tukey's tests indicated a decrease between baseline (km 0) and km 5 (*p* = .044, *g* = 0.49) and between baseline and km 10 (*p* = .044, *g* = 0.51), while no difference was observed between km 5 and km 10 (*p* = .987, *g* = 0.04) (Figure 2).

## Discussion

The primary objective of this study was to describe variation of passive stiffness of the lower limb in healthy runners throughout a 10 km race at a competitive pace. A secondary objective was to investigate specific changes of the various structures in lower limb, including PF, PT and MG. The main hypothesis of the study, i.e., that lower limb stiffness would decrease during the 10 k run, was not supported. Instead AT stiffness, as well as PT and MG, remained stable. In contrast, PF stiffness decreased by approximately 7% between the start of the race (0 km) and the 5th km, and this reduction persisted until the end of the run.

The lack of change in AT, MG, and PT stiffness suggests that these tissues may be more resilient to the mechanical demands of a 10 km run at moderate-to-high intensity. The study by Houghton et al. (20) reported that a 60 min treadmill run did not induce changes in the stiffness, estimated from the force displacement curve methods, of the AT but rather affected muscular output of ankle plantar flexor (20). With the same methods, Peltonen et al. (21) found no influence of a marathon on AT stiffness when measured one hour post-race (21). These conclusions align with other studies who observed no changes in AT stiffness after a 30-minute steady-state speed or incremental speed running session on a treadmill (3, 23). In the present study, running for one hour or less may not have provided sufficient mechanical stimulus to induce rheological change in the AT (7). Conversely, other studies have reported a decrease in passive stiffness of the AT following longer activities. Ooi et al. (24) observed a reduction in stiffness after a marathon, assessed using elastography (24). Similarly, Fletcher et al. (22) reported a decrease in AT stiffness following a 90 min run at 85% of lactate threshold, which was associated with changes in running economy (22). This reduction was attributed to greater tendon elongation, while maximum voluntary contraction remained constant. These discrepancies could also be due to some different study design, i.e., protocol and methods of stiffness measurement as well as participant characteristics. More specifically, the intensity of running pace, the training status, i.e., trained vs. novice, injured vs. healthy runners as well as the neuromuscular training added or not in the running regimen could influence passive stiffness response (3, 25). There was no study that had enough data to provide sufficient insight about the gender influence in stiffness variation during running.

A previous study found that stiffness of the medial gastrocnemius declined after an incremental running protocol to exhaustion (3), suggesting that fatigue-related mechanisms can alter passive muscle properties. In contrast, we did not observe such a reduction in the present study. This discrepancy may be explained by differences in running protocols and the associated fatigue state of the participants. Specifically, while the previous study used a maximal incremental test until exhaustion, our runners maintained a constant submaximal pace over 10 km. It is plausible that peripheral fatigability manifests differently under these conditions. During running, the ankle plantar flexors are repeatedly engaged in stretch–shortening cycles, which combine eccentric and concentric contractions in a unique pattern (31). This functional specificity may lead to different fatigue responses compared with isolated contractions (26), and could partly explain why muscle stiffness remained stable in the current protocol.

The selective decrease in PF stiffness raises intriguing questions. A previous work by Shiotani et al., who also reported a significant decrease in passive stiffness during a 10 km outdoor race on asphalt (27). They proposed mechanical fatigue and microscopic damage as plausible mechanisms. More recently, Krumpl et al. (28) extended these findings, showing that even brief bouts of high-intensity interval running induce acute reductions in PF stiffness and thickness, with only partial recovery after 30 min (28). These results indicate that the PF is highly responsive not only to prolonged, submaximal loading but also to short, intense efforts, underscoring the sensitivity of its mechanical properties across a wide spectrum of running demands. The PF's structural features likely contribute to this responsiveness. Its dense collagen network, although adapted to repetitive loading, may be particularly susceptible to fatigue when exposed simultaneously to tensile forces from the triceps surae and compressive loads at the calcaneal insertion during ground contact (29). Unlike tendons, which primarily transmit uniaxial tensile forces, the PF is subjected to both tensile forces from the triceps surae and compressive loads from ground contact, particularly at the calcaneal insertion.

Regarding our methodology, several aspects require improvement or modification for future studies or repetitions. Firstly, the running pace utilized in our study did not consistently induce a state of exhaustion among participants, contrary to our intention of simulating a competitive pace. Unfortunately, we did not assess the perceived intensity of the race at its conclusion using an effort intensity scale. Integrating such an assessment in future studies would be beneficial. To achieve this, we propose increasing the pace from the current range of 75%–85% of MAS to 85%–90%. However, it is important to acknowledge that increasing the pace carries the risk of participants being unable to maintain it, leading to a potential reduction in pace or cessation of the run. To address this concern, a stepwise approach could be adopted, incorporating a notion of failure and success to progress to the next step. In this framework, failure to maintain the prescribed pace or stopping altogether would signify the failure of the step and the conclusion of measurements for the participant. This approach offers a balanced compromise between achieving the desired intensity of the run and managing participant fatigue and adherence. The gender imbalance in the sample substantially limits the generalizability of the findings, suggesting that the present results may not extrapolate to female runners. It should be noted that PF stiffness was measured barefoot, whereas AT, MG, and PT stiffness were assessed while participants wore shoes. This discrepancy introduces a footwear-related confounding factor, limiting direct comparisons of PF responses with those of other tissues. Another important consideration is the relatively high standard deviations observed in stiffness values, i.e., around 12% to 15%, which reflect both biological variability and potential measurement error. Finally, no apriori sample size estimation was performed and therefore, external validity should be taken with caution. A posteriori power analysis considering multiple variables ranged from 0.5 for the Achilles tendon to 0.79 for the plantar fascia.

## Practical implication

The present findings demonstrate that repeated, in-field stiffness assessments using myotonometry are feasible during a 10 k run, enabling the monitoring of specific tissue responses such as those observed in the plantar fascia. However, the clinical relevance of stiffness changes remains uncertain. Assessing stiffness during running efforts and monitoring changes in stiffness during recovery could provide valuable insights into the progression and resolution of pathological conditions, such as tendinopathy or fasciapathy (30). Accordingly, the present findings should be considered hypothesis-generating rather than prescriptive for injury prevention.

## Conclusion

The present study rejected the initial hypothesis that Achilles tendon stiffness would decrease during a 10 km run, as AT, PT, and MG stiffness remained unchanged. In contrast, plantar fascia stiffness decreased by approximately 7%, suggesting a tissue-specific response to prolonged running. These findings underscore that lower limb structures do not adapt uniformly to running loads and highlight the PF as a structure of particular interest for future work on performance and injury risk.

## Data Availability

The raw data supporting the conclusions of this article will be made available by the authors, without undue reservation.
